# Royal Naval School

**Published:** 1831

**Authors:** 


Royal Naval School.

Our naval readers, and they are not very few, will be gratified to learn that a Royal Naval School, for the education of the sons of naval and marine officers, has been established under the patronage of His Majesty, and a large list of distinguished officers in the naval service. It is not a little remarkable, that the celebrated and Reverend Dr. A. Bell, Prebendary of Westminster, has given to this institution the magnificent donation of £20,000. in the three per cents, on the condition that the Madras system of education be adopted in the school—as exemplified in the Royal Military Asylum at Chelsea ! ! ! It is hardly necessary to state that this princely offer was instantly accepted, and that the institution is in rapid progress of organization. The object is to form a school for educating the sons of naval and marine officers (not below ward-room rank) at the lowest expense, and including board. All officers of and above ward-room rank, are eligible to become members of this institution, "on annually paying, *in advance*, one day's half-pay." All such members are to have a vote in the general meetings, with the power of nominating their children, as they attain the age of seven years, as candidates for admission into the school. These candidates are to be admitted in strict rotation, as vacancies occur, or as the establishment is enlarged.* All patriotic persons, by subscribing one guinea annually, are admitted as members, and have one vote in the general meetings. Donors of £100. have the absolute nomination of one boy (the son of an officer and member) to gratuitous board and education, for a period not exceeding five years, between the age of seven and fourteen years.

A further admission of gratuitous scholars is also contemplated, according as the funds may, on trial, be found to supply the means. Such gratuitous scholarships are to be reserved for the assistance either of orphans, or of members with large families; so that from the fund thus provided for gratuitous education, either the whole or part of the required board may be remitted, as particular cases may seem to require. It is proposed that the above gratuitous scholarships be entitled "The Dickson and Bell Scholarships," in acknowledgment of the merits of the originator of this scheme, and its bountiful benefactor.

We cannot but heartily wish this institution every success. The object is not only exceedingly good in itself, but it is peculiarly desirable in a period of peace, when a very large number of naval and marine officers are scarcely able to supply themselves and families with food, much less to bestow a liberal education on their sons.

